# Field suitability and diagnostic accuracy of the Biocentric® open real-time PCR platform for plasma-based HIV viral load quantification in Swaziland

**DOI:** 10.1186/s12879-018-3474-1

**Published:** 2018-11-14

**Authors:** Bernhard Kerschberger, Qhubekani Mpala, Paola Andrea Díaz Uribe, Gugu Maphalala, Roberto de la Tour, Sydney Kalombola, Addis Bekele, Tiwonge Chawinga, Mukelo Mliba, Nombuso Ntshalintshali, Nomcebo Phugwayo, Serge Mathurin Kabore, Javier Goiri, Sindisiwe Dlamini, Iza Ciglenecki, Emmanuel Fajardo

**Affiliations:** 1 Medecins Sans Frontieres (OCG), P.O. Box 18, Eveni, Lot No. 331, Sheffield Road, Industrial Area, Mbabane, Swaziland; 2 Ministry of Health (National Reference Laboratory), Mbabane, Swaziland; 3 Medecins Sans Frontieres (OCG), Geneva, Switzerland; 4 Clinton Health Access Initiative (CHAI), Mbabane, Swaziland; 5 Medecins Sans Frontieres (Access Campaign), Geneva, Switzerland

**Keywords:** HIV, Biocentric, Open platform, Viral load, Accuracy, Swaziland

## Abstract

**Background:**

Viral load (VL) testing is being scaled up in resource-limited settings. However, not all commercially available VL testing methods have been evaluated under field conditions. This study is one of a few to evaluate the Biocentric platform for VL quantification in routine practice in Sub-Saharan Africa.

**Methods:**

Venous blood specimens were obtained from patients eligible for VL testing at two health facilities in Swaziland from October 2016 to March 2017. Samples were centrifuged at two laboratories (LAB-1, LAB-2) to obtain paired plasma specimens for VL quantification with the national reference method and on the Biocentric platform. Agreement (correlation, Bland–Altman) and accuracy (sensitivity, specificity) indicators were calculated at the VL thresholds of 416 (2.62 log_10_) and 1000 (3.0 log_10_) copies/mL. Leftover samples from patients with discordant VL results were re-quantified and accuracy indicators recalculated. Logistic regression was used to compare laboratory performance.

**Results:**

A total of 364 paired plasma samples (LAB-1: *n* = 198; LAB-2: *n* = 166) were successfully tested using both methods. The correlation was high (*R* = 0.82, *p* < 0.01), and the Bland–Altman analysis showed a minimal mean difference (− 0.03 log_10_ copies/mL; 95% CI: -1.15 to 1.08). At the clinical threshold level of 3.0 log_10_ copies/mL, the sensitivity was 88.6% (95% CI: 78.7 to 94.9) and the specificity was 98.3% (95% CI: 96.1 to 99.4). Sensitivity was higher in LAB-1 (100%; 95% CI: 71.5 to 100) than in LAB-2 (86.4%; 95% CI: 75.0 to 94.0). Most upward (*n* = 8, 2.2%) and downward (*n* = 11, 3.0%) misclassifications occurred at the 2.62 log threshold, with LAB-2 having a 16 (95% CI: 2.26 to 113.27; *p* = 0.006) times higher odds of downward misclassification. After retesting of discordant leftover samples (*n* = 17), overall sensitivity increased to 93.5% (95% CI: 85.5 to 97.9) and 97.1% (95% CI: 90.1 to 99.7) at the 2.62 and 3.0 thresholds, and specificity increased to 98.6% (95% CI: 96.5 to 99.6) and 99.0% (95% CI: 97.0 to 99.8) respectively.

**Conclusions:**

The test characteristics of the Biocentric platform were overall comparable to the national reference method for VL quantification. One laboratory tended to misclassify VL results downwards, likely owing to unmet training needs and lack of previous hands-on practice.

**Electronic supplementary material:**

The online version of this article (10.1186/s12879-018-3474-1) contains supplementary material, which is available to authorized users.

## Background

The World Health Organization (WHO) recommends routine viral load (VL) testing at 6 and 12 months after initiation of antiretroviral therapy (ART) and annually thereafter [1]. Quantifying the patient’s VL allows clinicians to monitor the effectiveness of ART, to trigger adherence counselling interventions when VL is elevated above a clinical threshold (e.g. ≥1000 copies/mL), to diagnose virological failure, and to make timely and correct decisions on treatment switching [1–3]. Because the WHO recommends immediate initiation of ART at the time of HIV diagnosis irrespective of CD4 cell count and WHO staging criteria [1, 4], the number of patients needing routine VL testing will increase in the coming years. Although HIV programmes using routine VL monitoring have shown decreased morbidity and mortality [3], the expansion of VL testing creates clinical and programmatic challenges in resource-limited settings (RLS) [5, 6] and access to HIV monitoring services remains suboptimal [7, 8].

An important bottleneck is the suboptimal capacity of national laboratories in RLS to perform VL testing at scale. The supply weakness is often due to lack of funding to procure VL testing platforms and consumables, inability to recruit and retain qualified staff, lack of adequate training, and suboptimal servicing and maintenance of equipment [7]. Establishment of multiple laboratories in one country and the deployment of various platforms by different stakeholders (e.g. non-governmental organizations) is one strategy to overcome supply chain shortfalls and stimulate market competition [5]. This approach, however, raises concerns about comparability of VL test results between platforms and laboratories as well as about quality assurance and control.

Swaziland is increasing access to routine VL monitoring. The Ministry of Health performs VL testing using the Roche method, and Médecins Sans Frontières (MSF) has been performing VL quantification using the Biocentric method [9]. In 2015, the decision was taken to perform an in-country assessment of the Biocentric method to assess its suitability for contributing to expansion of VL testing in Swaziland. Thus, we compared the performance of the Biocentric platform under field conditions using plasma for VL testing in comparison with the national reference platform. The findings reported here are part of a larger prospective evaluation study comparing the test characteristics of the Biocentric platform, using different sampling and processing procedures (plasma and dried-blood spots [DBS]) for VL testing.

## Methods

### Setting

Swaziland is the country with the highest HIV prevalence (32% in people aged 18–49 years) in the world [10]. HIV care and treatment has been expanded, and close to 150,000 people received ART in 2015 [11]. Swaziland is expanding routine VL monitoring, and several VL platforms have been established. Three Roche platforms are operated, one at the National Reference Laboratory at Mbabane and two at decentralized sites (Manzini, Siteki). Since 2012, the Biocentric platform has been used in Nhlagano Laboratory in southern Swaziland, serving 25 rural primary and secondary healthcare facilities, with approximately 25,000 VL tests performed annually. It has been enrolled in the External Quality Assurance Program with the US Centers for Disease Control and Prevention (CDC) for proficiency testing. In addition, a second Biocentric platform was established at the National Reference Laboratory in 2016 but had not been used before this study. This study used the more recent Biocentric platform which was released in 2016. It was upgraded at Nhlangano laboratory (LAB-1) and newly installed at the National Reference Laboratory in Mbabane (LAB-2).

### VL platforms

The reference platform was the quantitative COBAS AmpliPrep/COBAS TaqMan (CAP/CTM) HIV-1 Test, Version 2.0 (Roche Molecular Diagnostics, Indiana, USA), operated at LAB-2 (Mbabane). It is a fully automated, closed system testing 63 samples per run with 5–8 h needed to obtain results. The lower limit of detection is 20 copies/mL (corresponding to 1.3 log_10_ copies/mL). Standardized internal quality control samples are provided and the reference laboratory is enrolled with the CDC laboratory external quality assurance program, monitoring the quality of VL testing and reporting twice per year.

The comparator comprised two Biocentric platforms operated at LAB-1 (Nhlangano) and at LAB-2 (Mbabane). This multi-manufacturer open platform consists of an open automated RNA and DNA extractor (Arrow®) and a real-time PCR system (FluoroCycler® 96) for nucleic acid amplification and detection. It uses the Generic HIV Charge Virale assay and test kits, which were developed by the French Agency for Research on AIDS and viral hepatitis (ANRS) and are manufactured and commercialized by Biocentric (Bandol, France) [12]. Internal quality control is provided by standards in the assay. This somewhat manual system has a time to results of approximately 3 h, with 96 samples per run (82 patient samples, five standards per duplicate, and one positive and one negative control per duplicate). The average limit of detection of HIV RNA at a positivity rate of > 95% with 250 μL plasma input volume is 416 (95% CI: 388 to 450) copies/mL [12]. The Biocentric assay received CE certification by a European Notified Body (British Standards Institution) and has been submitted for WHO pre-qualification of in vitro diagnostics. Further details on the method are available elsewhere [13].

### Study sample and procedures

**Fig. 1:**
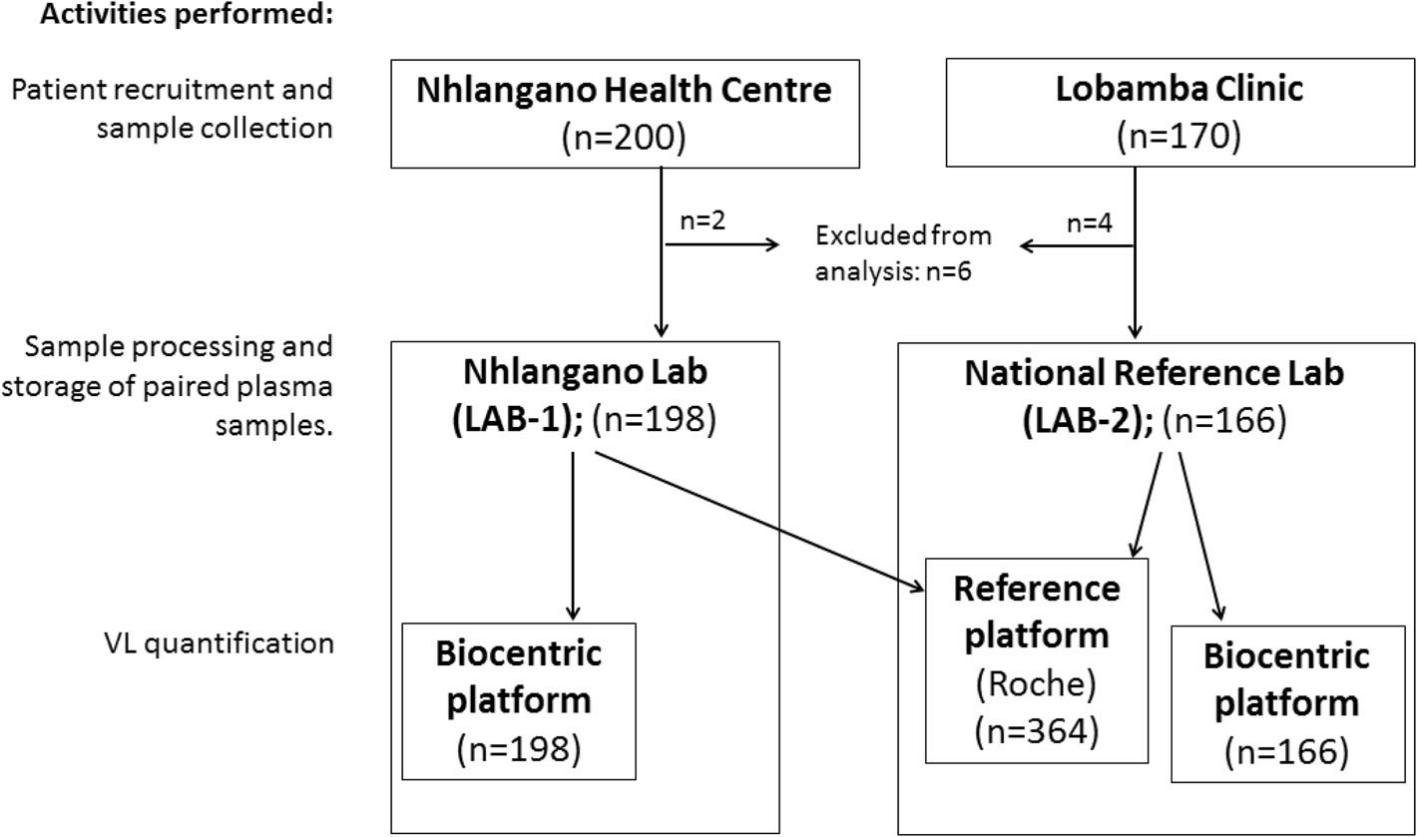
Study flow chart

Experienced laboratory technologists at LAB-1 received short refresher training on the Biocentric platform. Most laboratory technologists at LAB-2 had no experience in the Biocentric method and they received training over 3 days as per recommendation of the manufacturer. Figure 1 shows the study flow chart. From 12 October 2016 to 1 March 2017, HIV-infected adults (≥18 years) were recruited at Nhlangano Health Centre and Lobamba Clinic when they were eligible for VL testing according to the local VL testing algorithm (a baseline VL before ART initiation and during ART). During the recruitment phase, Lobamba Clinic introduced universal ART provision (thus many patients were eligible for ART initiation and received a pre-treatment VL test), while most patients in Nhlangano Health Centre were already established on ART (and thus received a follow-up VL test). The nurse obtained written consent, collected baseline information and referred patients for phlebotomy. A phlebotomist obtained one 4 mL venous blood ethylenediaminetetraacetic acid (EDTA) tube from each participant. In addition, a second EDTA tube and DBS cards were prepared as part of the larger study (details and results not reported here). The blood tubes obtained at Nhlangano Health Centre were sent to LAB-1 and those obtained at Lobamba Clinic to LAB-2.

In both laboratories, technologists centrifuged the EDTA tube to obtain two paired plasma specimens of 1 mL, which were stored in two separate sterile tubes at − 20 °C before testing. As the reference method was located at the National Reference Laboratory (and collocated to Biocentric LAB-2), deep frozen plasma samples were shipped (2 h) from LAB-1 to LAB-2 for testing on the reference platform. All testing runs were performed with a plasma input volume of 250 μL on the Biocentric method and 1 mL on the Roche method. VL results that were discrepant between the two methods at LAB-1 and LAB-2 were repeated on the Biocentric method in the same laboratory when leftover plasma samples were available. The laboratory personnel were blinded to the results of both methods.

### Statistical analysis

This study is reported according to the STARD guidelines [14]. Patients without a plasma test result on both platforms were removed from analysis. Baseline characteristics of the study population were described and summarized in frequency statistics and percentages. To compare baseline characteristics of patients by recruitment site, differences in continues (e.g. age) and categorical (e.g. sex) data were assessed with the Wilcoxon rank sum test and the Pearson’s chi-squared test. We regarded the VL results from the reference method (CAP/CTM) as the national gold standard. Because the two assays had different lower and upper detection limits, VL test results were equalized at the common lowest (2.62 log_10_ copies/mL) and highest reliable (7.0 log_10_ copies/mL) detection limits. We assessed the correlation between the two methods graphically and with the Pearson’s correlation coefficient for quantifiable VL values ≥2.62 log_10_ copies/mL on the two platforms. Then we used Bland–Altman analysis to describe agreement between the two platforms by calculating the mean difference along with 95% limits of agreement [15]. Sensitivity and specificity were calculated using the threshold of 2.62 log_10_ copies/mL (lower limit of detection, corresponding to 416 copies/mL) and 3.0 log_10_ copies/mL (clinical threshold, corresponding to 1000 copies/mL). The positive and negative predictive values (PPV and NPV) were computed assuming 10 and 20% VL elevations in a hypothetical population undergoing VL testing. All analyses were conducted separately for each laboratory and both laboratories combined.

In sensitivity analyses, to account for prolonged turnaround times from sample collection to freezing of paired plasma samples, diagnostic accuracy estimates (sensitivity, specificity) were recalculated for samples with processing times of ≤4.0 h. In addition, misclassified values were described separately at the patient level and accuracy estimates recalculated after re-quantification of discordant VL results. Discordance was defined as VL results which were categorized differently by the Biocentric platform (above or below) compared with the reference test, using a binary VL cut-off at 2,62 and 3.0 log_10_ copies/mL. Because LAB-2 appeared to have had higher rates of misclassification, we evaluated a possible association between laboratory (LAB-2 vs LAB-1) and VL result misclassification. Potential confounding factors were identified a priori using directed acyclic graphs (DAGs) [16] and included in multivariable penalized maximum likelihood logistic regression models. All analyses were performed with STATA v14.1 (StataCorp, Texas, USA).

## Results

### Baseline characteristics

We recruited 370 patients, of whom six (1.6%) were excluded from analysis: three were less than 18 years of age and three had insufficient or sub-optimal quality plasma samples for VL quantification (Fig. 1). Of the remaining 364 patients with paired VL testing results available (Table 1), the median age was 36 (interquartile range [IQR]: 30–44.5) years, 231 (64.7%) and 15 (4.2%) were non-pregnant and pregnant women respectively, and 305 (83.8%) patients received a VL test while on ART (median time on ART 5.0 (IQR 2.0–7.5) years). Nhangano Health Centre recruited 198 (54.4%) patients who, compared with Lobamba Clinic, were more likely to be men (32.5% vs 29.4%) and non-pregnant women (67.0% vs 61.9%), were older (39 vs 32.5 years), were more likely to have received a VL test during ART (98.0% vs 66.9%) and had been on ART for longer (6.2 vs 2.9 years). All samples from Nhlangano Health Centre (*n* = 198) were sent for processing to LAB-1 and all samples from Lobamba Clinic (*n* = 166) to LAB-2 (Fig. 1). The median time from EDTA collection to plasma storage at − 20 °C (processing time) was 1.9 (IQR: 1.1–3.3) hours, and it was shorter for LAB-1 (1.2, IQR: 0.9–1.9) than for LAB-2 (3.1, IQR: 2.2–4.1) (*p* < 0.01). Overall, 54 (14.8%) samples were stored for between 4 and 6 h, and one sample for 6.9 h. The median time from freezing of the plasma sample to testing on the reference and Biocentric platforms was 21.5 (IQR: 13–28) and 89 (IQR: 56–103) days respectively.

**Table 1:** Baseline characteristics of the study population by recruitment site/ laboratory and overall

	Both facilities combined	Nhlangano (LAB-1)^a^	Lobamba (LAB-2)^a^	*p*-value
Total	364	198 (54.4)	166 (45.4)	
Age; median (IQR), years	36 (30–44.5)	39 (33–48)	32.5 (27–39)	< 0.01
Gender and pregnancy status (missing = 7)				< 0.01
Men	111 (31.1)	64 (32.5)	47 (29.4)	
Non-pregnant women	231 (64.7)	132 (67.0)	99 (61.9)	
Pregnant women	15 (4.2)	1 (0.5)	14 (8.8)	
Reason for VL test				< 0.01
Pre-ART	59 (16.2)	4 (2.0)	55 (33.1)	
ART	305 (83.8)	194 (98.0)	111 (66.9)	
Time on ART; median (IQR), years	5.0 (2.0–7.5)	6.2 (3.3–8.3)	2.9 (1.8–5.4)	< 0.01
VL values on the reference method; log_10_ copies/mL				< 0.01
< 1.3	236 (64.8)	150 (75.8)	86 (51.8)	
1.3–< 3.0	58 (15.9)	37 (18.7)	21 (12.7)	
3.0–< 4.0	17 (4.7)	5 (2.5)	12 (7.2)	
≥ 4.0	53 (14.6)	6 (3.0)	47 (28.3)	

*ART* Antiretroviral therapy, *IQR* Interquartile range, *VL* Viral load

^a^VL samples obtained in Nhlangano Health Centre were tested at LAB-1 (Nhlangano), and VL samples obtained in Lobamba Clinic were tested at LAB-2 (Mbabane)

### Results of VL quantification using the reference method

According to the reference method, 236 (64.8%) specimens had a VL below the detection limit, and 58 (15.9%) had a VL of 1.3–< 3.0, 17 (4.7%) of 3.0–< 4.0 and 53 (14.6%) of ≥4.0 log_10_ copies/mL. The median VL of specimens with detectable VLs (*n* = 128) on the reference method was 3.42 (IQR: 1.66–4.91) log_10_ copies/mL. LAB-1 received more undetectable (< 1.3 log_10_ copies/mL) paired specimens (*n* = 150, 75.8%) than LAB-2 (*n* = 86, 51.8%; *p* < 0.01), and the median VL among detectable measurements was also lower (LAB-1: 1.57, IQR: 1.3–2.76; LAB-2: 4.26, IQR: 2.91–5.10; *p* < 0.01) (Table 1).

### Correlation and agreement

**Fig. 2:**
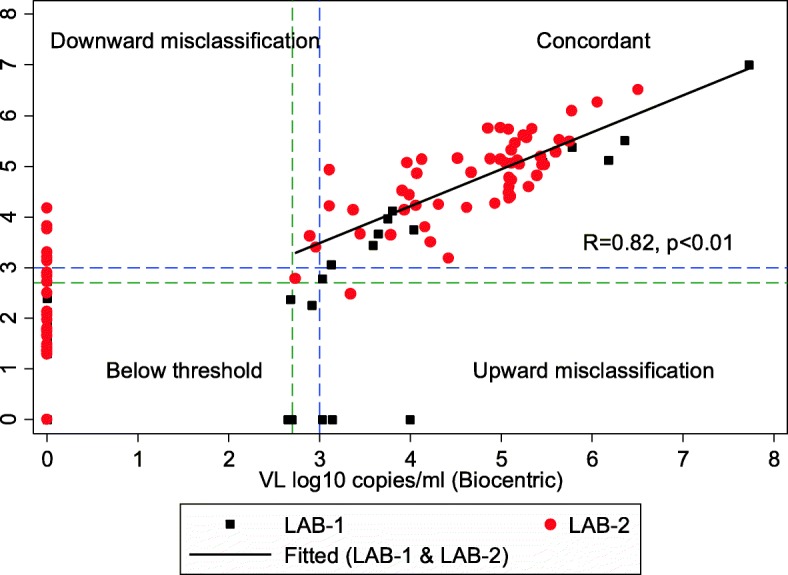
Assay correlation and concordance between the Biocentric platform and the reference method. VL, viral load; R, Pearson’s correlation coefficient; p, *p*-value; LAB-1, laboratory 1 in Nhlangano; LAB-2, laboratory 2 in Mbabane. The correlation graph shows paired VL values obtained from the reference and Biocentric platforms. The Pearson’s correlation coefficient and the fitted linear regression line were calculated for quantifiable VL values above the threshold level of 2.62 log10 copies/mL on both methods (*n* = 66)

**Fig. 3:**
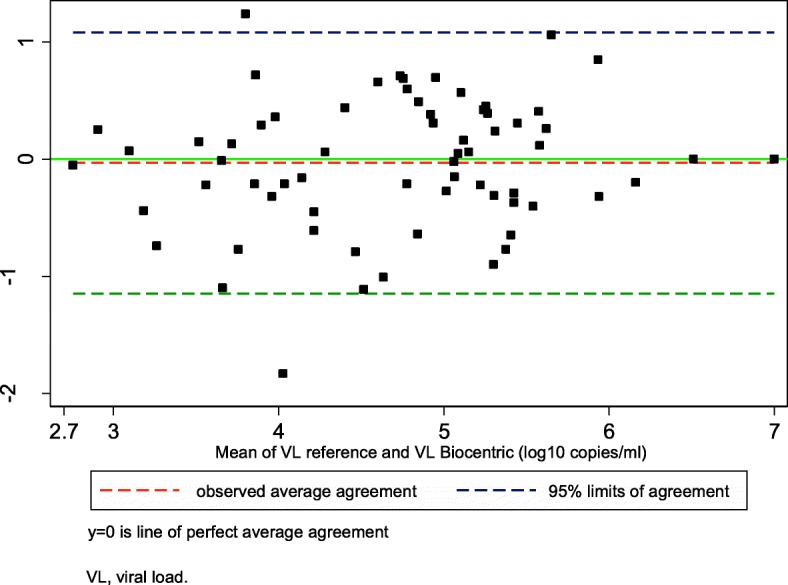
Bland–Altman mean difference analysis between the Biocentric platform and the reference method (n = 66). The analysis was performed for paired samples with a VL ≥2.62 log_10_ copies/mL on both the Biocentric and the reference platform

The Pearson’s correlation coefficient for quantifiable VL values above the threshold level of 2.62 log_10_ copies/mL on both methods (*n* = 66) showed a strong positive correlation between the reference method and Biocentric (*R* = 0.82, p < 0.01) and appeared higher in LAB-1 (*R* = 0.98, *p* < 0.01) compared with LAB-2 (*R* = 0.75, *p* < 0.01) (Fig. 2). Figure 3 shows the Bland–Altman difference plots for quantifiable VL results (*n* = 66) on both methods. The overall mean difference was minimal at − 0.03 (95% CI: -1.15 to 1.08) log_10_ copies/mL. It was 0.24 (95% CI: -0.54 to 1.03) log_10_ copies/mL for LAB-1 and -0.09 (95% CI: -1.24 to 1.05) log_10_ copies/mL for LAB-2. All values were within ±1.0 log_10_ copies/mL from the mean, and 60 (90.9%) were within ±0.5 log_10_ copies/mL from the mean.

### Diagnostic accuracy

Accuracy was calculated at two threshold levels (2.62 and 3.0 log_10_ copies/mL), and findings are presented in Table 2. The overall accuracy of the Biocentric platform was excellent at the 2.62 log threshold, with an area under the receiver operating characteristic (ROC) curve of 0.92 (95% CI: 0.87 to 0.96), and was similar for the two laboratories (LAB-1: 0.94, 95%CI: 0.87 to 1.00; LAB-2: 0.92, 0.87 to 0.96).

**Table 2:** Test characteristics of the Biocentric platform at two VL threshold levels

	At 2.62 log_10_ copies/mL			At 3.0 log_10_ copies/mL		
	LAB-1 (*n* = 198)	LAB-2 (*n* = 166)	Combined (*n* = 364)	LAB-1 (*n* = 198)	LAB-2 (*n* = 166)	Combined (*n* = 364)
Sensitivity % (95% CI)	92.3 (64.0–99.8)	84.4 (73.1–92.2)	85.7 (75.9–92.6)	100 (71.5–100)	86.4 (75.0–94.0)	88.6 (78.7–94.9)
Specificity % (95% CI)	96.2 (92.4–98.5)	99.0 (94.7–100)	97.2 (94.6–98.8)	97.9 (94.6–99.4)	99.1 (94.9–100.0)	98.3 (96.1–99.4)
ROC area % (95% CI)	0.94 (0.87–1.00)	0.92 (0.87–0.96)	0.92 (0.87–0.96)	0.99 (0.98–1.00)	0.93 (0.88–0.97)	0.93 (0.90–0.97)
PPV (at 10%)^a^ % (95% CI)	73.1 (56.3–85.1)	90.5 (57.6–98.5)	77.4 (63.2–87.2)	83.9 (66.3–93.2)	91.1 (59.3–98.6)	85.3 (70.7–93.3)
NPV (at 10%)^a^ % (95% CI)	99.1 (94.5–99.9)	98.3 (97.0–99.0)	98.4 (97.3–99.1)	100 (93.3–100)	98.5 (97.2–99.2)	98.7 (97.6–99.3)
PPV (at 20%)^a^ % (95% CI)	85.9 (74.4–92.8)	95.6 (75.3–99.3)	88.5 (79.4–93.9)	92.1 (81.6–96.9)	95.9 (76.6–99.4)	92.9 (84.5–96.9)
NPV (at 20%)^a^ % (95% CI)	98.0 (88.4–99.7)	96.2 (93.5–97.8)	96.5 (94.0–97.9)	100 (86.1–99.9)	96.7 (93.9–98.2)	97.2 (94.7–98.5)

*ROC* Receiver operating characteristic, *PPV* Positive predictive value, *NPV* Negative predictive value

^a^For the calculation of predictive values, 10 and 20% prevalence of detectable VLs were assumed in a hypothetical population undergoing routine VL testing

For the threshold levels of 2.62 and 3.0 log_10_ copies/mL, the overall (both laboratories combined) sensitivity was 85.7% (95% CI: 75.9 to 92.6) and 88.6% (78.7 to 94.9) respectively, and the specificity was 97.2% (94.6 to 98.8) and 98.3% (96.1 to 99.4). Although the specificity was similar in both laboratories, ranging from 96.2 to 99.1% at both threshold levels, the sensitivity was lower in LAB-2 at both log thresholds (at 2.62 log_10_ copies/mL: 84.4%, 73.1 to 92.1) compared with LAB-1 (at 2.62 log_10_ copies/mL: 92.3%, 64.0 to 99.8) (Table 2). While the sensitivity at the 3.0 log threshold was high at 100% (71.5 to 100) in LAB-1, it remained low in LAB-2 (86.4%, 75.0 to 94.0). At the 2.62 log threshold, the combined PPV was 77.4 (63.2 to 87.2) and the NPV was 98.4 (97.3 to 99.1) assuming a prevalence of 10% VL elevation, and 88.5 (79.4 to 93.9) and 96.5 (94.0 to 97.9) respectively assuming a prevalence of 20%. Both the PPV and NPV remained similar when calculated at the 3.0 log threshold.

In sensitivity analyses, the sensitivity and specificity estimates at both VL log thresholds remained similar after removal of samples with > 4.0 h (*n* = 55) or missing (*n* = 1) processing times (2.62 log threshold: sensitivity 85.7% (74.6 to 93.3), specificity 96.7 (93.7 to 98.6); 3.0 log threshold: sensitivity 89.5% (78.5 to 96.0), specificity (98.0% (95.4 to 99.4)).

### Misclassification

At the threshold of 2.62 log_10_ copies/mL, 19/364 (5.2%) samples were misclassified: 11/364 (3.0%) samples were misclassified downwards and 8/364 (2.2%) were misclassified upwards (Table 3). Among these, five samples were below the lower detection limit of the reference method but were detected on the Biocentric platform, and 11 samples were quantified on the reference method but not detected on the Biocentric platform. Misclassification occurred across all quantification levels of the reference method: five in the VL range of < 1.3 log_10_ copies/mL, eight in the range of 1.3–< 3.0 log_10_ copies/mL, five in the range of 3.0–< 4.0 log_10_ copies/mL, and one at ≥4.0 log_10_ copies/mL. Of note, 57.9% (*n* = 11) of misclassification occurred in LAB-2, of which 10/11 (90.9%) were downward misclassifications. Overall, 18/19 (94.7%) discordant samples differed more than 0.5 log_10_ copies/mL at the threshold of 2.62 log_10_ copies/mL and 11/13 (84.6%) at the threshold of 3.0 log_10_ copies/mL.

**Table 3:** Original and reclassified viral load test results between the Biocentric platform and the reference assay

Lab	Reason for VL testing	Time to freezing (hours)^a^	VL results during first round of VL quantification				VL results after re-quantification		
			Reference method (log_10_ copies/mL-l)	Biocentric platform (log_10_ copies/mL-l)	Misclassification		Biocentric platform (log_10_ copies/mL-l)	Misclassification	
					2.62 log_10_ copies/mL	3.0 log_10_ copies/mL		2.62 log_10_ copies/mL	3.0 log_10_ copies/mL
Lab-1	ART	2.7	0	3.14	upward	upward	*	*	*
Lab-1	ART	3.8	0	2.65	upward	CON	0	CON	CON
Lab-1	ART	1.1	0	3.03	upward	upward	0	CON	CON
Lab-1	ART	0.9	0	4.00	upward	upward	0	CON	CON
Lab-1	ART	0.5	0	2.70	upward	CON	*	*	*
Lab-1	ART	0.3	2.25	2.92	upward	CON	*	*	*
Lab-1	ART	1.5	2.37	2.68	upward	CON	0	CON	CON
Lab-2	Pre-ART	2.2	2.48	3.34	upward	upward	2.78	upward	CON
Lab-2	ART	4.4	2.72	0	downward	CON	0	downward	CON
Lab-1	ART	1.6	2.73	0	downward	CON	*	*	*
Lab-1	ART	2.4	2.78	3.03	CON	upward	*	*	*
Lab-2	Pre-ART	2.6	2.84	0	downward	CON	2.84	CON	CON
Lab-2	Pre-ART	1.2	2.90	0	downward	CON	0	downward	CON
Lab-2	Pre-ART	1.4	2.91	0	downward	CON	3.00 (999^b^)	CON	upward^b^
Lab-2	Pre-ART	1	3.13	0	downward	downward	0	downward	downward
Lab-2	ART	2.6	3.21	0	downward	downward	3.51	CON	CON
Lab-2	Pre-ART	3	3.31	0	downward	downward	0	downward	downward
Lab-2	Pre-ART	3.2	3.40	2.96	CON	downward	3.84	CON	CON
Lab-2	Pre-ART	4.7	3.63	2.89	CON	downward	3.60	CON	CON
Lab-2	Pre-ART	4.1	3.77	0	downward	downward	3.06	CON	CON
Lab-2	ART	0.3	3.82	0	downward	downward	3.95	CON	CON
Lab-2	Pre-ART	2.5	4.17	0	downward	downward	4.29	CON	CON

*Re-quantification on the Biocentric platform was not possible as no leftover plasma samples were available due to contamination. Reclassification of test results was not performed

Zero values indicate that the VL results were below the detection limit of the VL assays

*CON* Concordant

^a^Time from sample collection to freezing at − 20 °C before testing

^b^Due to rounding, the 3.00 log_10_ copies/mL values represent a false-positive test result at the 3.0 log_10_ copies/mL threshold but a concordant result according to the non-log_10_ values

After adjustment for potential factors associated with misclassification (see Additional file 1), multivariate analysis showed that LAB-2 had a 15.99 (95% CI: 2.26 to 113.27; *p* = 0.002) higher odds of downward misclassification at the 2.62 log threshold compared with LAB-1. No associations were found for the overall probability of discordant VL values (upward and downward misclassification combined), for upward misclassification or at the 3.0 log threshold level. The full regression model is presented in Additional file 2.

### Re-quantification of discordant VL values

Discordant VL values at both log thresholds were re-quantified on the Biocentric platform in corresponding LAB-1 and LAB-2 by the more experienced laboratory technologists when leftover samples were available. At the 2.62 log threshold, 15/19 (78.9%) leftover samples were re-quantified, of which 10 samples became concordant, one remained misclassified upwards (2.78 log_10_ copies/mL on the Biocentric platform vs 2.48 log_10_ copies/mL on the reference method) and four remained misclassified downwards (undetectable on the Biocentric platform vs 2.72, 2.90, 3.13 and 3.31 log_10_ copies/mL on the reference method). Re-quantification at the 3.0 log threshold yielded similar findings. Among the 11/13 (84.6%) successfully re-quantified results, nine VL values became concordant while two remained misclassified downwards (undetectable on the Biocentric platform vs 3.13 and 3.31 log_10_ copies/mL on the reference method). When we considered the re-quantified values and kept the original VL values for non-retested samples, the overall sensitivity estimates increased to 93.5 (95% CI: 85.5 to 97.9) and 97.1% (90.1 to 99.7) at the 2.62 and 3.0 thresholds, and specificity estimates increased to 98.6 (96.5 to 99.6) and 99.0% (97.0 to 99.8) respectively (Table 4). The PPV and NPV increased to 96.0% (88.5–98.7) and 99.3% (97.2–99.8) respectively when a prevalence of 20% VL elevation at the 3.0 log threshold was assumed.

**Table 4:** Test characteristics of the Biocentric platform (both laboratories combined) after re-quantification of discordant VL samples

	At 2.62 log_10_ copies/mL (*n* = 364)	At 3.0 log_10_ copies/mL (*n* = 364)
Sensitivity % (95% CI)	93.5 (85.5–97.9)	97.1 (90.1–99.7)
Specificity % (95% CI)	98.6 (96.5–99.6)	99.0 (97.0–99.8)
ROC area % (95% CI)	0.96 (0.93–0.99)	0.98 (0.96–1.0)
PPV (at 10%)^a^ % (95% CI)	88.2 (73.8–95.2)	91.4 (77.4–97.0)
NPV (at 10%)^a^ % (95% CI)	99.3 (98.3–99.7)	99.7 (98.8–99.9)
PPV (at 20%)^a^ % (95% CI)	94.4 (86.4–97.8)	96.0 (88.5–98.7)
NPV (at 20%)^a^ % (95% CI)	98.4 (96.3–99.3)	99.3 (97.2–99.8)

If VL re-quantification was not feasible, the first VL testing result was taken into account

*ROC* Receiver operating characteristic, *PPV* Positive predictive value, *NPV* Negative predictive value

^a^For the calculation of predictive values, 10 and 20% prevalence of detectable VLs were assumed in a hypothetical population undergoing routine VL testing

## Discussion

Improved access to VL monitoring is crucial in RLS to meet the fast growing monitoring needs of large ART cohorts. One strategy is the deployment of multiple platforms by different stakeholders. This study is the first in Swaziland and, to our knowledge, the second internationally [13] to evaluate the utility of the Biocentric platform using plasma for VL quantification under routine conditions in comparison with another method. We showed that the Biocentric platform performs reliably under routine conditions. It had a strong positive correlation with the reference method (*R* = 0.81, *p* < 0.01), and the overall agreement between the two methods was high (mean difference − 0.03) at the 3.0 log threshold. Although 5.2% of samples were misclassified at the threshold of 2.62 log_10_ copies, most discrepancies were resolved after re-quantification of discordant results, and the sensitivity and specificity increased to 97.1 and 99.0% at the 3.0 log_10_ VL threshold. These estimates were similar to those reported previously, where the sensitivity and specificity were 100 and 90% respectively compared with the HIV Amplicor Monitor assay (Roche Diagnostics, Basel, Switzerland) [13].

Misclassification of results occurred across all quantification levels and most of them with an absolute difference of more than 0.5 log_10_ copies/mL. This may indicate that misclassifications were due to factors beyond the technical variation of the platforms (e.g. operator differences). This study also showed inter-laboratory differences. Sensitivity was decreased in LAB-2, and LAB-2 emerged as an independent risk factor for downward misclassification (false negative) compared with LAB-1. Differences in quality between laboratories were likely due to manual sample preparation and reagent volume pipetting errors by staff who were less trained and experienced in this method. The Biocentric platform was newly established in LAB-2 and the training provided before the evaluation may have been insufficient. Disadvantages of this platform are that it is a manual technique requiring experienced staff, who cannot always be easily found or retained in RLS, and that manual techniques may be more prone to error [5, 6]. Therefore, intra- and inter-laboratory quality assurance mechanisms should be established (in addition to the internal controls provided by the assay) to detect suboptimal performance as soon as possible. As a consequence, the National Reference Laboratory decided to provide further formal and hands-on training before the routine use of this platform in LAB-2. Of note, inter-laboratory differences independent of the VL assay and differences between platforms were also reported in other settings [17, 18]. Because of the inherent variability between VL platforms, it is recommended that patients be monitored using the same technology platform to ensure correct interpretation of VL changes over time [19].

### Context specific considerations

When VL testing is introduced into routine settings, viral (e.g. genetic diversity of HIV strains), programmatic, laboratory-specific and clinical (e.g. definitions of viral failure) factors need to be taken into account to establish a contextualized VL testing strategy. Firstly, a positive aspect of this platform is its ability to be implemented in RLS, performing reliably under routine conditions specifically at the clinical threshold level of 3.0 log_10_ copies/mL. In our experience, maintenance requirements of this open platform are minimal and individual elements are interchangeable, such as RNA extraction techniques [20] and previously validated real-time PCR thermal cyclers [21]. Another positive factor is its high throughput volume. Four of the Biocentric-experienced laboratory technologists were able to perform up to three runs per day (246 tests per day) with four extractors and one thermal cycler under routine conditions.

Secondly, the use of plasma for VL quantification limits its use to settings with strong sample transportation systems in place and/or the capacity to prepare and store samples at clinical sites. According to Biocentric, DBS samples can also be used on the platform, requiring less logistical and cold-chain support. VL quantification on DBS cards on Biocentric is being evaluated in Swaziland and will be reported in future. Thirdly, the Biocentric platform is a polyvalent technology, which allows testing of VL in conditions other than HIV, such as HIV early infant diagnosis (EID) and hepatitis C VL. This is becoming increasingly important for programmes wishing to integrate laboratory services using multi-disease platforms [22].

Fourthly, the Biocentric HIV VL test is priced competitively (ex-works USD14.9 per test) compared with other well-established VL technologies [23]. Finally, the Biocentric VL reagents, as with other VL technologies, contain guanidine thiocyanate (GTC), which is a toxic chemical compound [24] commonly used for the extraction of DNA and RNA in molecular tests [25]. As GTC can release cyanide gases in contact with bleach and due to its toxicity to aquatic life, it has to be managed as hazardous waste, normally through high-temperature incineration [25]. This can pose logistical challenges in RLS and requires proper planning and budgeting.

### Limitations and strengths

A limitation of the study is that discrepant test results were not fully investigated. They were also not re-quantified on both methods owing to insufficient leftover plasma samples, with retesting being performed solely on the Biocentric platform. Although retesting of discordant results is not standard of practice in laboratory evaluation studies, retesting was performed to obtain additional information of the nature of discrepant results, assuming that the suboptimal performance of LAB-2 was likely due to less hands-on practice of the laboratory technologists rather than problems with the Biocentric method itself. After retesting, a few samples remained discrepant, for which several explanations exist. Firstly, there is the possibility of false test results on the national reference platforms due to internal quality issues or operator errors. However, internal and external quality control did not indicate quality issues during the study period. Nevertheless, a third VL assay should have been used to resolve discrepant results. Secondly, the two platforms used different plasma input volumes, increasing the likelihood of variations in measurements for values at the detection threshold. Thirdly, transportation and storage conditions may have affected the sample quality, possibly leading to a degradation of RNA. Lastly, we did not test for HIV genotypic diversity. VL assays differ in their ability to quantify genetically diverse HIV strains, largely depending on the design of primers and probes [13, 26–29]. The CAP/CTM HIV-1 v2.0 detects HIV-1 groups M, N and O, and Biocentric detects HIV-1 group M (A–H) [28]. Without a panel of samples with genetic diversity, generalizability is limited, specifically to settings where other strains are endemic. However, according to a recent study in Cameroon, Biocentric performed well in that setting which is characterized by broad HIV genetic variability [30]. Another limitation is that the majority of VL samples were below the detection limit of the Biocentric platform, reducing the sample size for correlation and Bland–Altman analyses. Finally, we did not assess reproducibility. This study focused on field diagnostic accuracy and is not a pure analytical study. Repeat testing would have been complex to undertake at various conditions (intra and inter-variability) because it would have required more VL samples from patients.

A strength of the study was its conduct under routine real-world conditions; therefore, challenges and constraints are comparable to other RLS in Sub-Saharan Africa. Also, the personnel involved from sample collection (phlebotomist) to VL testing (laboratory technologists) are likely to reflect staff composition of other RLS.

## Conclusions

The Biocentric platform using plasma for VL quantification showed results that were comparable overall to the national reference method. This study also revealed inter-laboratory differences in performance, which was likely due to unmet training needs and lack of hands-on practice of technologists in one laboratory, highlighting the need for continuous training of laboratory personnel. In addition to participation in national and international proficiency testing programmes, routine quality control methods should be integrated into laboratories performing at high scale in RLS to detect suboptimal performance as soon as possible. The Biocentric platform is now routinely used in Swaziland to support the expansion of VL testing.

## Additional files

Additional file 1: Directed acyclic graph (DAG) presenting possible relationships between Biocentric laboratory (LAB-1/LAB-2) and the probability of VL misclassification. (PDF 194 kb)

Additional file 2: Multivariable penalized maximum likelihood logistic regression models of risk factors associated with misclassification. (PDF 181 kb)

## Abbreviations

ART: Antiretroviral therapy
DBS: Dried-blood spots
EDTA: Ethylenediaminetetraacetic acid
LAB-1: Laboratory 1
LAB-2: Laboratory 2
NPV: Negative predictive value
PPV: Positive predictive value
RLS: Resource limited setting
VL: Viral load
WHO: World Health Organization
