# Supplementary material for: Field suitability and diagnostic accuracy of the Biocentric® open real-time PCR platform for plasma-based HIV viral load quantification in Swaziland
Source: BMC Infect Dis. 2018 Nov 14;18:570. doi: 10.1186/s12879-018-3474-1 (PMC6236955; doi:10.1186/s12879-018-3474-1)
Supplement: Supplementary file 1 — Directed acyclic graph (DAG) presenting possible relationships between Biocentric laboratory (LAB-1/LAB-2) and the probability of VL misclassification. (PDF 194 kb) [file 12879_2018_3474_MOESM1_ESM.pdf]

Additional file 1: Directed acyclic graph (DAG) presenting possible relationships between Biocentric laboratory (LAB-1/LAB-2) and the probability of VL misclassification.

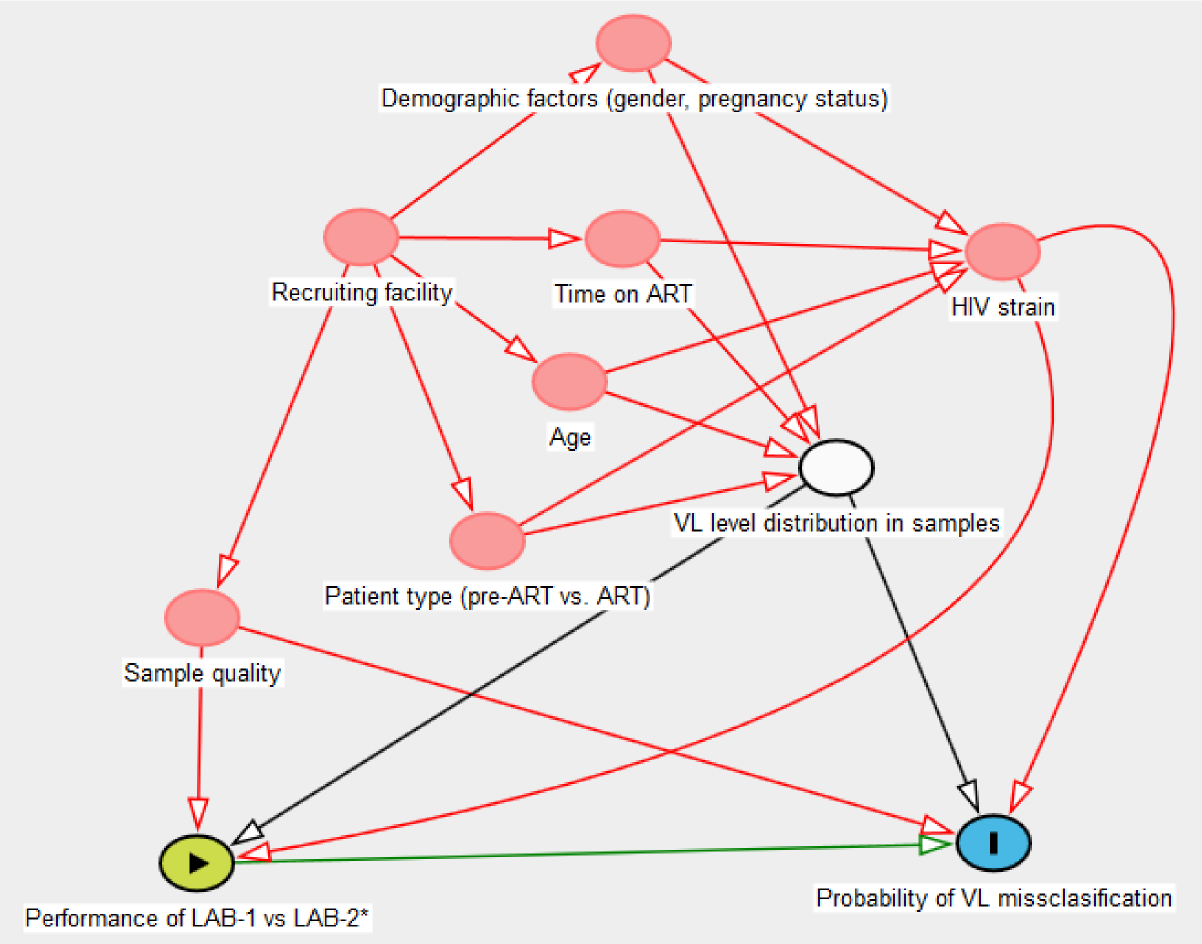

The directed acyclic graph (DAG) was created with the DAGitty browser based application for generating, editing and analysing directed acyclic graphs [1,2]. The assumptions were as follows: The intervention assumed to be associated with the likelihood of VL misclassifications was the overall performance ability of each Biocentric laboratory (LAB-1 vs LAB-2) to perform the VL test correctly – which was likely predicted by laboratory specific factors such as (unmet) trainings needs and previous hands-on experience. Other factors may have confounded this association. We assumed that recruiting facility was associated with characteristics of study participants (age, patient type, time on ART, gender and pregnancy status) which predicted VL measurement level distribution and potentially VL strain diversity in plasma samples. The non-random distribution of VL measurement levels and VL strains may predict the ability to correctly perform the VL tests. For instance, reagents volume adequacy errors by less experienced staff may more likely lead to false test results if the true VL levels are close to the lower detection threshold of the Biocentric assay. In addition, sample quality may predict performance ability to conduct the test without error as well as the probability of misclassification. According to the DAG, the minimal sufficient adjustment to estimate the association

between performance ability of the laboratory and the outcome are sample quality, VL strain distribution and VL measurement values distribution in samples. We adjusted for VL distribution in the regression model. Sample quality could not be measured directly but was partly adjusted for through the proxy variable of time from sample collection to freezing/ storage. The variable viral strain distribution was not adjusted for as no information on strain diversity was available.

References

1. DAGitty - drawing and analyzing causal diagrams (DAGs) [Internet]. [cited 2017 May 31]. Available from: http://www.dagitty.net/

2. Textor J, Hardt J, Knüppel S. DAGitty: a graphical tool for analyzing causal diagrams. Epidemiol Camb Mass. 2011;22:745.
