# Supplementary material for: Field suitability and diagnostic accuracy of the Biocentric® open real-time PCR platform for plasma-based HIV viral load quantification in Swaziland
Source: BMC Infect Dis. 2018 Nov 14;18:570. doi: 10.1186/s12879-018-3474-1 (PMC6236955; doi:10.1186/s12879-018-3474-1)
Supplement: Supplementary file 2 — Multivariable penalized maximum likelihood logistic regression models of risk factors associated with misclassification. (PDF 181 kb) [file 12879_2018_3474_MOESM2_ESM.pdf]

Additional file 2: Multivariable penalized maximum likelihood logistic regression models of risk factors associated with misclassification.

At 2.62 log_{10} copies/ml									
	**Discordant**			**Upward misclassification**			**Downward misclassification**		
	**aOR**	**95% CI**	**p**	**aOR**	**95% CI**	**p**	**aOR**	**95% CI**	**p**
**Biocentric laboratory**									
LAB-1	1			1			1		
LAB-2	2.81	(0.87 - 9.13)	0.085	0.35	(0.04 - 2.93)	0.331	15.99	(2.26 - 113.27)	0.006
**VL results[1], log_{10} copies/ml**									
<1.3	1			1			1		
1.3-<3.0	6.89	(2.25 - 21.10)	0.001	2.61	(0.66 - 10.31)	0.171	48.73	(2.59 - 916.07)	0.009
3.0-<4.0	14.75	(3.81 - 57.17)	0.000	1.69	(0.09 - 33.50)	0.730	123.37	(6.21 - 2,450.60)	0.002
≥4.0	0.85	(0.13 - 5.66)	0.863	0.74	(0.03 - 16.57)	0.847	5.36	(0.21 - 136.53)	0.309
**Sample preparation time, hours**	0.71	(0.45 - 1.10)	0.127	0.99	(0.53 - 1.86)	0.974	0.65	(0.37 - 1.12)	0.122

At 3.0 log_{10} copies/ml									
	**Discordant**			**Upward misclassification**			**Downward misclassification**		
	**aOR**	**95% CI**	**p**	**aOR**	**95% CI**	**p**	**aOR**	**95% CI**	**p**
**Biocentric laboratory**									
LAB-1	1			1			1		
LAB-2	2.33	(0.51 - 10.77)	0.277	2.33	(0.51 - 10.77)	0.277	2.33	(0.51 - 10.77)	0.277
**VL results[1], log_{10} copies/ml**									
<1.3	1			1			1		
1.3-<3.0	2.88	(0.56 - 14.97)	0.207	2.88	(0.56 - 14.97)	0.207	2.88	(0.56 - 14.97)	0.207
3.0-<4.0	35.90	(8.57 - 150.38)	0.000	35.90	(8.57 - 150.38)	0.000	35.90	(8.57 - 150.38)	0.000
≥4.0	1.31	(0.17 - 9.92)	0.792	1.31	(0.17 - 9.92)	0.792	1.31	(0.17 - 9.92)	0.792
**Sample preparation time[2], hours**	0.87	(0.52 - 1.45)	0.586	0.87	(0.52 - 1.45)	0.586	0.87	(0.52 - 1.45)	0.586

[1] These viral load results were obtained from the reference platform (Roche).

[2] Time from whole blood sample collection until plasma preparation and storage.

aOR, adjusted odds ratio; VL, viral load.

Multivariable penalized maximum likelihood logistic regression models were built to assess if laboratory (LAB-1 vs LAB-2) was associated with overall, downward and upward misclassification of VL testing values at the thresholds of 2.62 and 3.0 log_{10} copies/ml. Potential predictors of misclassification for inclusion into the models were identified a priori through directed acyclic graphs
